# A new high-throughput screening-compatible gap junctional intercellular communication assay

**DOI:** 10.1186/s12896-015-0211-3

**Published:** 2015-10-06

**Authors:** Ju Yeon Lee, Eun Ju Choi, Jinu Lee

**Affiliations:** College of Pharmacy, Yonsei Institute of Pharmaceutical Sciences, Yonsei University, 85 Songdogwahak-ro, Yeonsu-gu, Incheon 406-840 South Korea

**Keywords:** Gap junction, High-throughput screening, Iodide, YFP

## Abstract

**Background:**

Gap junctions (GJs) are intercellular channels through which molecules smaller than 1 kDa can diffuse, and they have been suggested as drug targets. To develop chemical drugs acting on this target, a high-throughput screening (HTS) system for GJ modulators is necessary.

**Results:**

We designed a new, high-throughput GJ intercellular communication (GJIC) assay. This assay system consisted of donor and acceptor cells from LN215 glioma cells that expressed SLC26A4 and yellow fluorescent protein-H148Q/I152L (YFP^QL^), respectively. The fluorescence of LN215-YFP^QL^ acceptor cells, when cultured alone, was not quenched by iodide. However when donor and acceptor cells, or LN215-YFP^QL^ and LN215-I^−^ cells, were mixed and plated, they formed GJs. When iodide was added, it was transported into donor cells by SLC26A4, diffused through the GJs to acceptor cells, and quenched the YFP^QL^ fluorescence. The quenching rate was optimal at a 2:1 mixture of donor and acceptor cells. The assay quality parameter, Z’ factor, was calculated from data collected with vehicle and carbenoxolone. For each assay, the Z’ factor increased with time. The Z’ factor of a 10-s assay was 0.72 indicating that the assay quality was high enough for use in HTS. This assay system also worked well in HOS osteosarcoma cells with a Z’ factor at 10 s of 0.70.

**Conclusions:**

We developed a new HTS system for GJ modulators. The system had a high assay quality with a Z’ factor ≥ 0.70, was rapid and required only 10 s per well, was inexpensive in requiring no additional reagents, and was predicted to have a low rate of false-positive hits.

**Electronic supplementary material:**

The online version of this article (doi:10.1186/s12896-015-0211-3) contains supplementary material, which is available to authorized users.

## Background

Gap junctions (GJs) are intercellular channels that allow diffusion of small molecules (<1 kDa) including nutrients, metabolites, and signalling molecules such as cAMP, Ca^2+^, and IP_3_ [1]. Six connexins form a tube-like structure called a connexon, which works as a hemichannel that opens to the extracellular space or can dock to another connexon on a neighbouring cell, forming a GJ. The human connexin family contains 21 connexin proteins named for their molecular weights [2].

The functional importance of connexins is substantiated by various diseases that are caused by mutations in connexin genes. Mutations in *GJB2*, which encodes human connexin26, lead to syndromic hearing loss with skin symptoms or non-syndromic hearing loss [3]. Disruptions of connexin43 by mutations in *GJA1* result in oculodentodigital dysplasia causing multiple developmental abnormalities [4].

There is also evidence that GJ is a pharmacologic target in disease. GJs play a crucial role in propagating cardiac muscle contractions. Thus, GJ modulators have therapeutic efficacy for arrhythmia. AAP, a naturally occurring anti-arrhythmic peptide, exerts its activity by enhancing GJ [5]. Its orally-active derivative, GAP134, prevents post-operative atrial fibrillation. GJs can spread cell death signals or harmful molecules from cells in a damaged area to relatively healthy cells and, thus, aggravate tissue damage. A GJ inhibitor, 2-aminoethoxydiphenyl borate (2-APB), was shown to protect against liver damage caused by hepatotoxic drugs [6]. Pharmacologic inhibition of the connexin43 hemichannel with Gap26 protects cardiomyocytes *in vivo* [7]. The therapeutic effects of bisphosphonates on osteoporosis are partially due to their anti-apoptotic effects mediated by binding to the connexin43 hemichannel [8].

To assess GJ activity, dye transfer-based methods have been used [9]. Lucifer yellow is frequently used as a GJ-permeable fluorescent dye and is introduced into cells by microinjection [10], scrape loading [11], or electroporation [12]. GJ activity is determined by quantifying diffusion of the dye into cells without the dye. Another dye, calcein-AM (acetoxymethyl ester) has been used for a fluorescence recovery after photobleaching assay [13]. In this assay, cells are preloaded with calcein-AM before cells of interest are bleached with a high-power laser beam. Calcein diffusion from non-bleached cells to bleached cells is measured by time-lapse confocal imaging. Another method to assess GJ activity is measurement of junctional conductance using a dual patch clamp. In contrast to dye transfer, it has high sensitivity and high temporal resolution. However, because micropipettes are inserted into two adjacent cells, and the surfaces are sealed before measuring junctional conductance, the dual patch clamp method is technically demanding and time-consuming [9]. To develop drugs targeting GJ, a high-throughput screening (HTS)-compatible assay system is needed. However applying the methods described above to HTS is practically impossible.

Here we developed and report a simple, inexpensive, rapid, robust (shown by Z’ factor), and HTS-compatible GJIC assay.

## Results

### Design of I^−^-YFP^QL^ GJIC assay

**Fig. 1:**
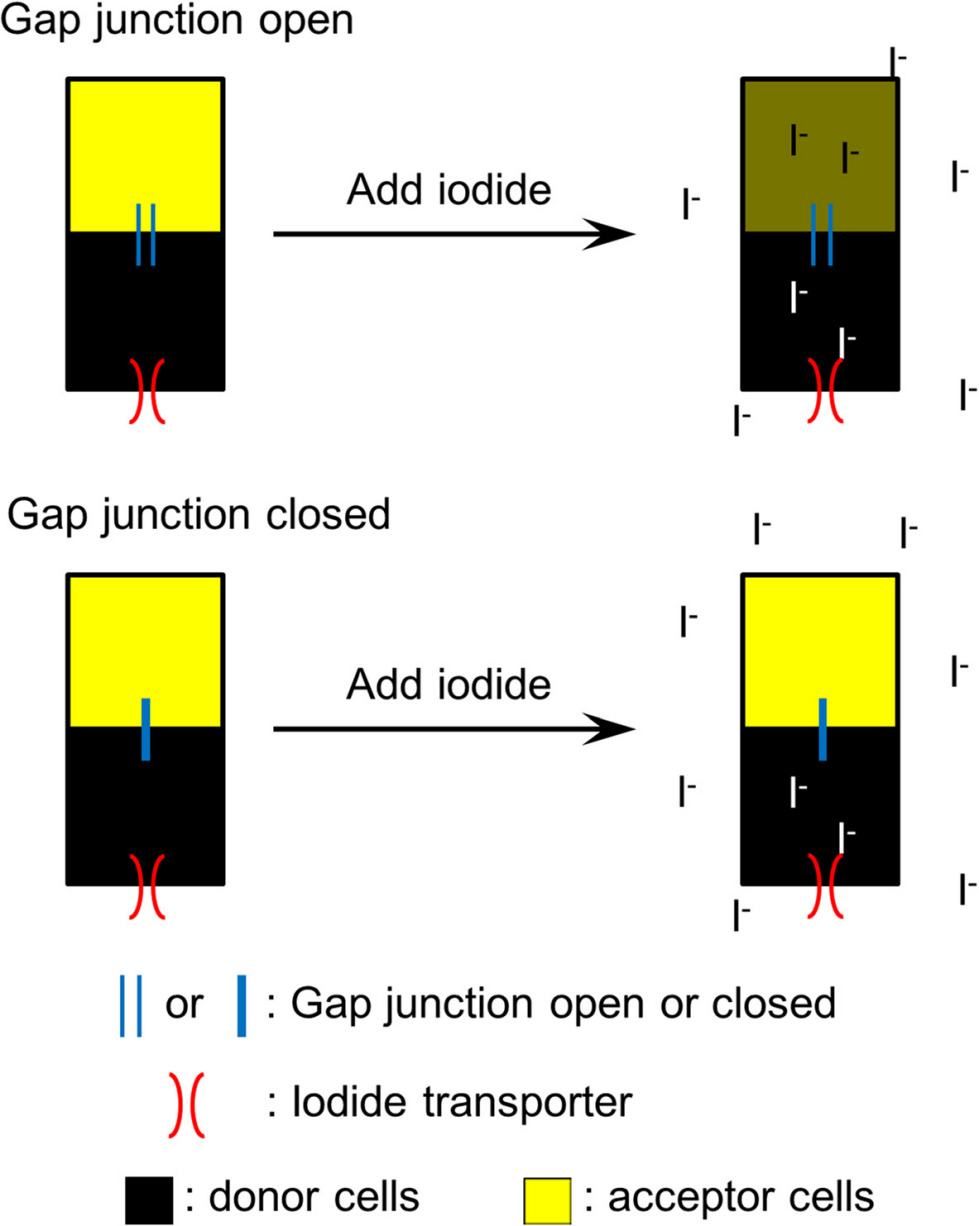
The scheme of I^−^-YFP^QL^ GJIC assay. Black and yellow rectangles represent donor cells expressing an iodide transporter, SLC26A4, and acceptor cells with YFP^QL^. Blue and red parallel lines indicate gap junctions and iodide transporters, respectively. Acceptor cells were impermeable to iodide. Added iodide could only reach acceptor cells by passing through the iodide transporter in the donor cells and then through the gap junction between donor and acceptor cells. The YFP^QL^ in acceptor cells was quenched by the iodide

We designed a new GJ intercellular communication (GJIC) assay that used two types of cells (Fig. 1). Donor cells expressed a membrane protein capable of transporting extracellular iodide into cells. Acceptor cells expressed an iodide sensor protein, yellow fluorescent protein H148Q/I152L (hereafter YFP^QL^) [14]. When iodide was added to a co-culture of the two cells, whose cytosols were connected by GJs, the iodide entered the donor cells, passed through the GJs to acceptor cells, and quenched the fluorescence of YFP^QL^. If GJs were absent or closed, the added iodide diffused into donor cells but not acceptor cells, and YFP^QL^ fluorescence did not change.

### I^−^-YFP^QL^ GJIC assay in the co-culture of LN215-I^−^ and LN215-YFP^QL^

**Fig. 2:**
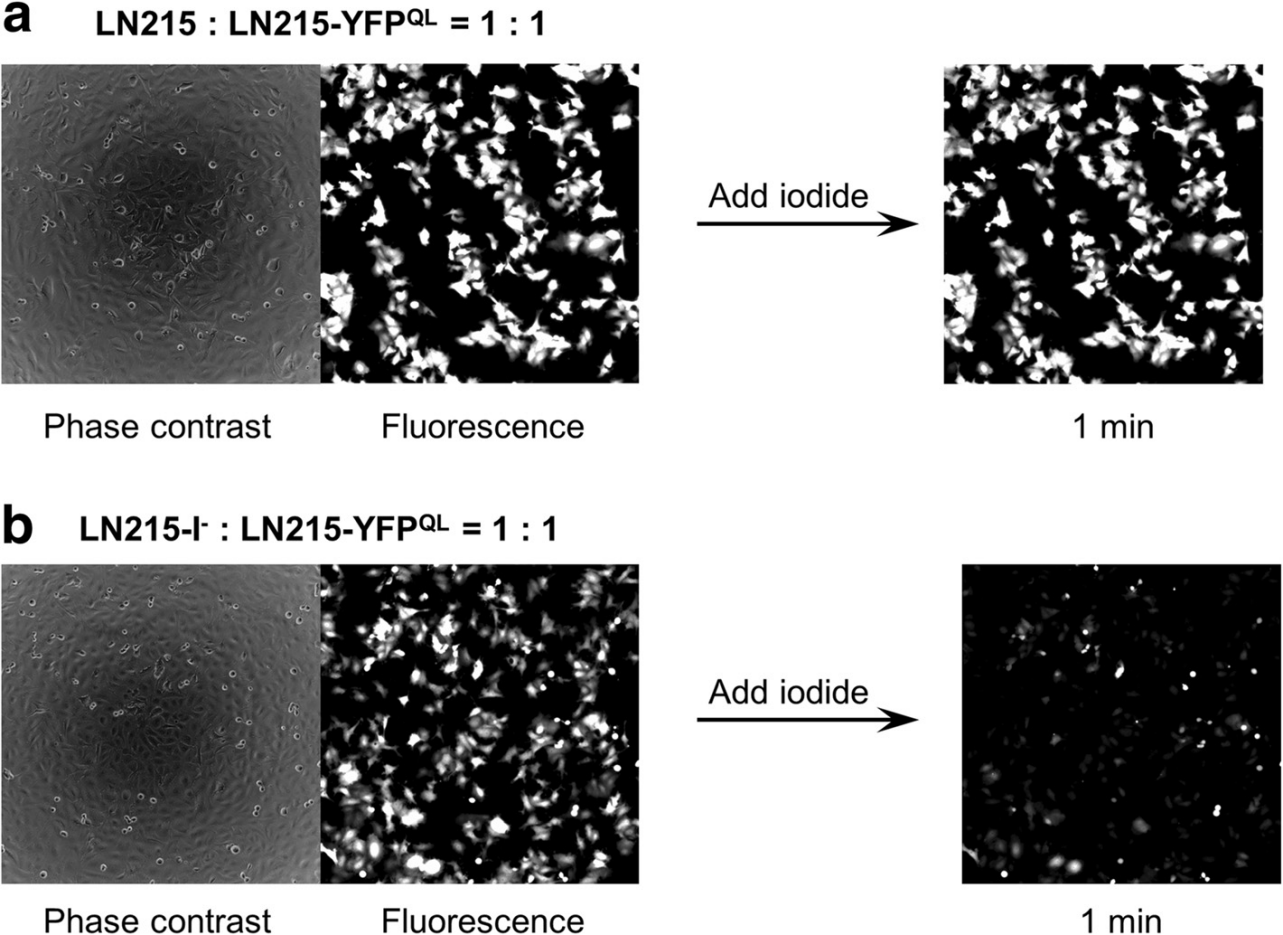
Fluorescence image of I^−^-YFP GJIC assay in LN215 cells. The LN215-YFP^QL^ cells combined with wild-type LN215 (**a**) or LN215-I^-^ (**b**) cells were plated on a 96-well plate. After 24 h of incubation, the culture media were replaced with 100 μL of C-solution. Phase contrast and fluorescence images were collected before and 1 min after adding 100 μL of I-solution. For the composition of the C- and I-solutions, see the I^−^-YFP^QL^ GJIC assay in the Methods section

To generate cells expressing enough iodide transporter or YFP^QL^, we produced lentiviruses expressing SLC26A4 or YFP^QL^. LN215, a human glioma cell, was chosen as the target cell type because astrocytes and glioma cells express functional GJs [15]. We also confirmed through immunoblotting that LN215 cells express connexin43 (Additional file 1). We generated LN215-I^−^ donor cells and LN215-YFP^QL^ acceptor cells through lentiviral transduction followed by antibiotic selection. LN215-YFP^QL^ cells combined with wild-type LN215 cells or LN215-I^−^ cells were plated on 96-well plates 24 h before the assay. Culture media were changed to C-solution, a balanced salt solution without iodide, and fluorescence images were collected before and 1 min after adding I-solution, a balanced salt solution containing 140 mM iodide. As shown in Fig. 2a, the YFP^QL^ fluorescence of wells containing wild-type LN215 and LN215-YFP^QL^ cells did not decrease 1 min after addition of I-solution. Conversely, the fluorescence of the wells with LN215-I^−^ and LN215-YFP^QL^ cells decreased when iodide was added (Fig. 2b). These results suggest that iodide did not enter LN215-YFP^QL^ cells directly; however, when LN215-I^−^ donor and LN215-YFP^QL^ acceptor cells were plated in the same well, iodide entered LN215-I^−^ and traveled through GJs to LN215-YFP^QL^.

### Optimizing the I^−^-YFP^QL^ GJIC assay

**Fig. 3:**
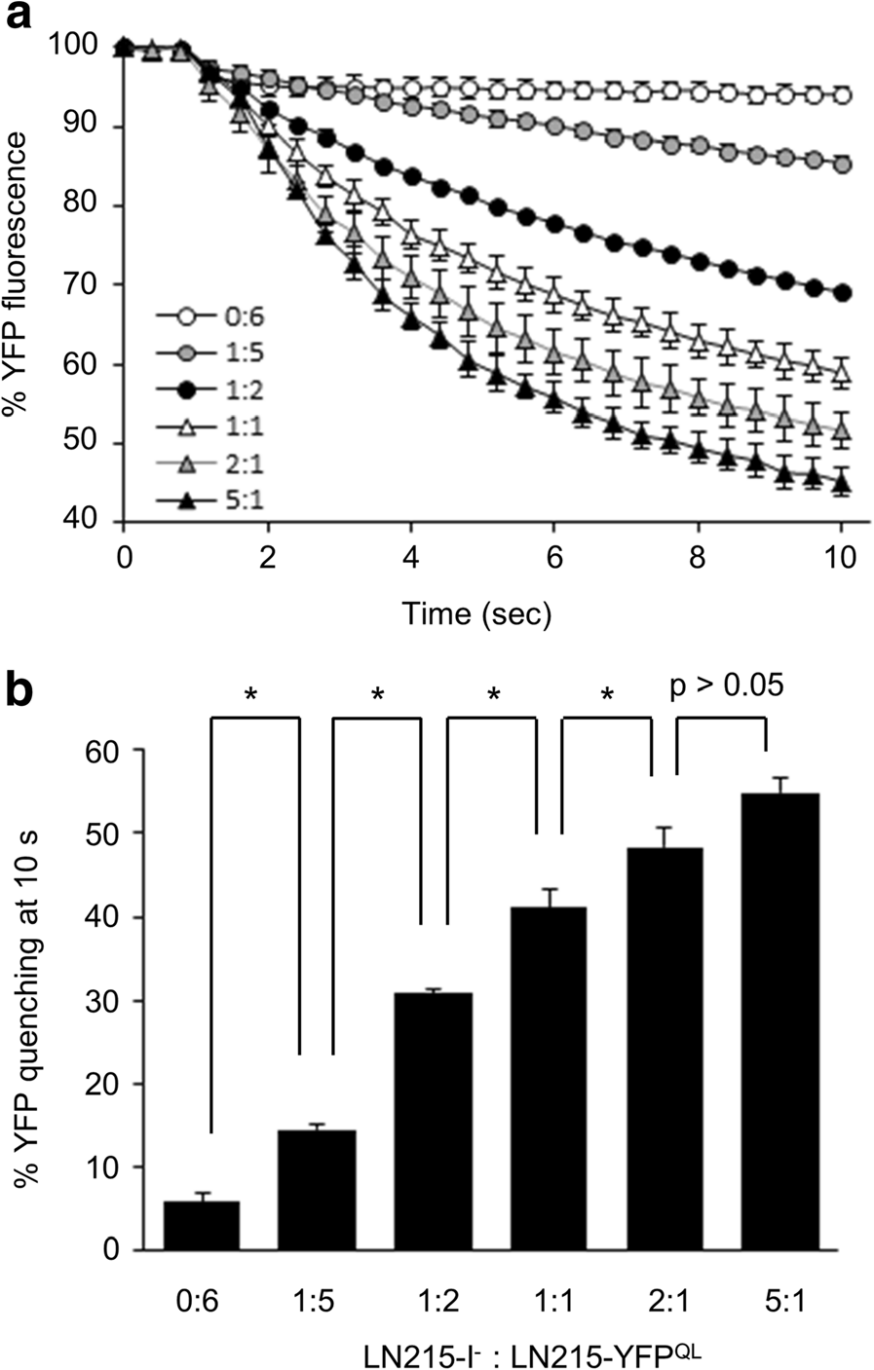
Determination of the optimal ratio of donor and acceptor cells. LN215-I^-^ and LN215-YFP^QL^ cells were mixed and plated on 96-well plates at ratios of 0:6, 1:5, 1:2, 1:1, 2:1, 5:1 and 6:0 and incubated for 24 h. After culture media were replaced with 100 μL of C-solution, the plate was inserted into the multi-plate reader. The YFP^QL^ fluorescence of each well was read every 0.4 s for 10 s. One second after the first measurement, 100 μL of I-solution was added by the automatic injector in the plate reader. Assay of each group was performed in three wells. The mean fluorescence intensity of the 6:0 group at each time point was considered as background and subtracted from fluorescence intensities of the other groups at the corresponding time point. Then, the relative YFP fluorescence at each time was normalized to the fluorescence of the starting point and plotted against time. Error bars represent standard deviations (**a**). The percent final YFP quenching was calculated and expressed as mean ± standard deviation (**b**). *, *p* < 0.05 (Student *t*-test)

To determine the optimal ratio of donor and acceptor cells for the GJIC assay, we plated 0:6, 1:5, 1:2, 1:1, 2:1, 5:1, and 6:0 mixtures of donor and acceptor cells before the GJIC assay. The mean fluorescence intensities of wells with donor cells only (6:0) at each time point were subtracted from fluorescence intensities of the other groups at the corresponding time point before calculation of % YFP fluorescence. As shown in Fig. 3a, the fluorescence of wells with acceptor cells only (0:6) did not decrease when iodide was added. The fluorescence of wells with mixed cells decayed with time after adding iodide. The final quenching rates were shown as a bar graph (Fig. 3b). The larger was the number of donor cells plated, the higher was the final quenching rate (GJIC activity). The quenching rates of neighboring two groups were significantly different (*p* < 0.05, Student’s *t*-test) except when 2:1 (48.3 ± 2.3 %, mean ± standard deviation) and 5:1 (54.8 ± 1.7 %) were compared. The 5:1 ratio might be better for obtaining a strong signal, but it takes more time to cultivate the number of cells needed to plate the two types of cells at 5:1 compared to that needed at 2:1. Thus for convenience, a 2:1 ratio of donor and acceptor cells was used in the later GJIC assays. The 10-s % YFP quenching with or without background subtraction was 48.3 and 42.1 %, respectively. Hence, to simplify data processing, background subtraction was omitted in the following data analysis. The raw fluorescence values (Additional file 2) without background subtraction or normalization to the starting points were also plotted against time and are presented as Additional file 3.

### Performance of the I^−^-YFP^QL^ GJIC assay

**Fig. 4:**
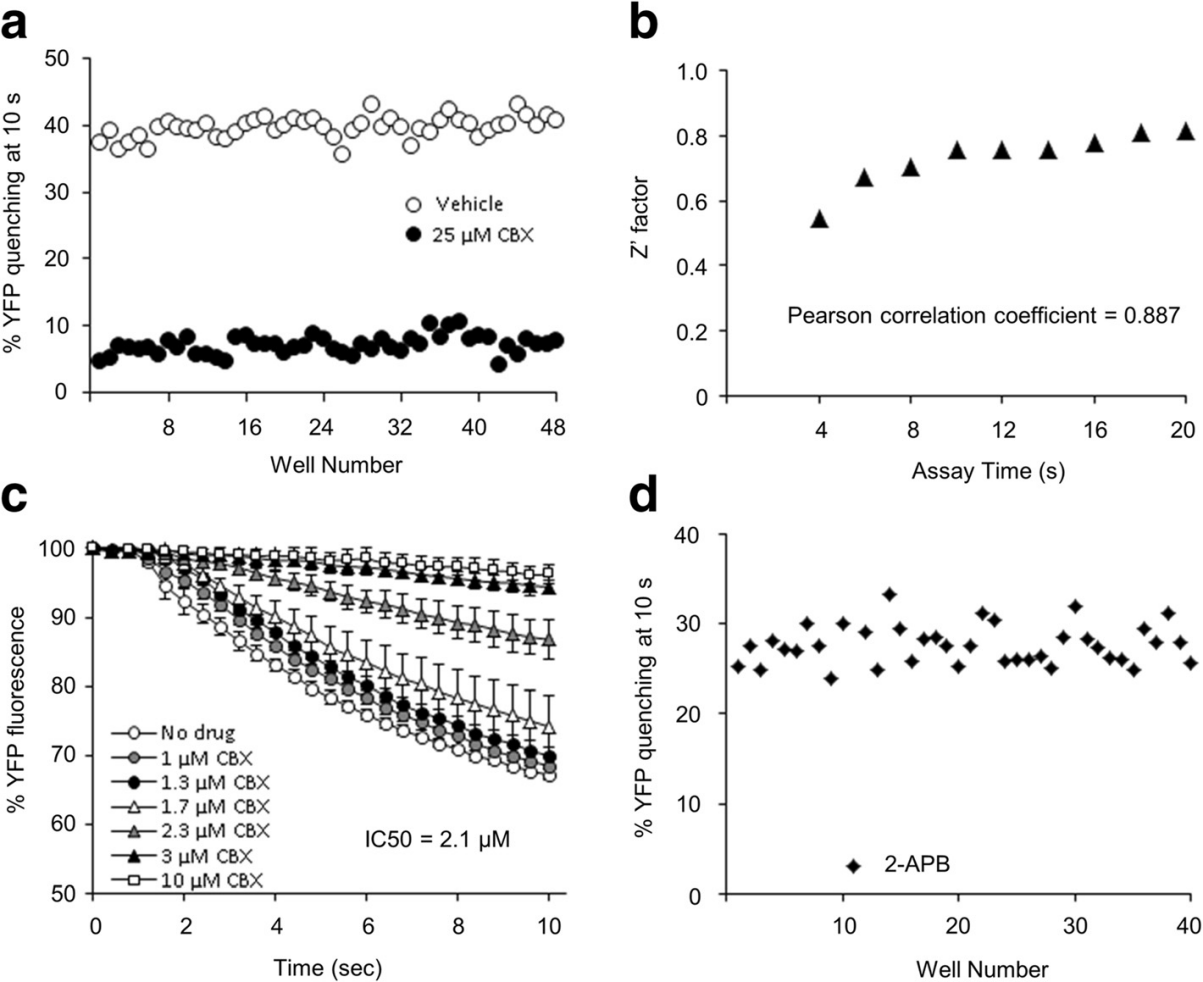
Performance of I^−^-YFP GJIC assay as a HTS system. To assess the quality of the I^−^-YFP GJIC HTS assay, a 2:1 mixture of LN215-I^-^ and LN215-YFP^QL^ cells was plated on a 96-well plate. After 24 h of cultivation, vehicle (water) or 25 μM of CBX diluted in C-solution was added to the well for 10 min, with each treatment being applied to 48 wells. To investigate the effect of assay time on *Z*’ factor, the I^−^-YFP assay was conducted for 20 s per well. The percent YFP quenching at 4, 6, 8, …, and 20 s was calculated. The values at 10 s were plotted against well number (**a**). Z’ factors were calculated using positive (CBX) and negative (vehicle) control data at each time point, as described in the Methods section, and the results were plotted against assay time. Pearson’s correlation coefficient was calculated in Microsoft Excel 2010 using Pearson’s function, which was 0.887 (**b**). The mixed cultures were treated with CBX at 0, 1, 1.3, 1.7, 2.3, 3 and 10 μM in C-solution for 10 min before the GJIC assay. Each treatment was added to five wells. The mean % YFP fluorescence was plotted against time. Error bars represent standard deviations. The IC_50_ for GJIC activity by CBX was calculated with GraphPad Prism 4 (GraphPad Software) (**c**). The 40 chemicals listed in Additional file 5 were applied to the mixed cultures before the GJIC assay. All chemicals were applied at a concentration of 25 μM. The % YFP quenching at 10 s was plotted against chemical number. All GJIC activities were within the range of the mean ± standard deviation except when 2-APB was applied (**d**)

To assess the performance of the LN215-I^−^/LN215-YFP^QL^ system as an HTS GJIC assay, we measured the effects of vehicle (water) and carbenoxolone (CBX), a widely used GJ inhibitor, on GJ activity as quantified by percent YFP^QL^ quenching. CBX was applied at 25 μM, at which concentration a 30-min treatment was not toxic to LN215 cells (data not shown) and GJIC was completely inhibited [16]. The quenching rates of all well at 10 s were plotted against well number and are shown in Fig. 4a. The mean ± standard deviation of vehicle and the CBX groups were 42.9 ± 1.8 and 7.3 ± 1.3 %, respectively. The Z’ factor has been used as an assay quality indicator, reflecting the signal window between positive and negative controls [17]. Z’ factors were calculated from % YFP quenching at 4, 6, 8… 20 s and plotted against assay time. As the assay was prolonged, the YFP quenching rates increased and the quality parameters also increased from 0.54 at 4 s to 0.81 at 20 s. Because an HTS assay with a Z’ factor higher than 0.5 is considered acceptable, the I^−^-YFP^QL^ GJIC assay is robust enough to be used in an HTS [17]. In addition, to verify that the reduced quenching by CBX was the result of reduced GJ coupling rather than a direct effect of the drug itself, only LN215-YFP^QL^ cells or a 2:1 mixture of LN215-I^−^ and LN215-YFP^QL^ cells were plated 24 h before treatment with vehicle or 25 μM CBX for 10 min. Then, the I^−^-YFP^QL^ assay was performed for 10 s. The % YFP fluorescence was plotted against time and is shown in Additional file 4. When only the acceptor cells were plated, the CBX treatment did not produce a significant change in YFP fluorescence, which supported the supposition that CBX reduction in YFP quenching is due to its inhibition of GJs.

To assess the sensitivity of the I^−^-YFP^QL^ assay as a screening tool for GJIC modulator, we applied CBX to the mixed cultures at 0, 1, 1.3, 1.7, 2.3, 3 and 10 μM diluted in C-solution for 10 min before the I^−^-YFP^QL^ GJIC assay. These results are shown in Fig. 4c. The IC_50_ value for CBX was 2.1 μM with a 95 % confidence interval of 2.0–2.2 μM, which was similar to a previous report [16]. We measured the effects of 40 known chemicals at concentrations of 25 μM on GJIC activity using this screening system. One chemical, 2-APB, was found to act as a GJ inhibitor (Fig. 4d). The tested chemicals are listed in Additional file 5.

### Applying the I^−^-YFP^QL^ GJIC assay to another cell type

**Fig. 5:**
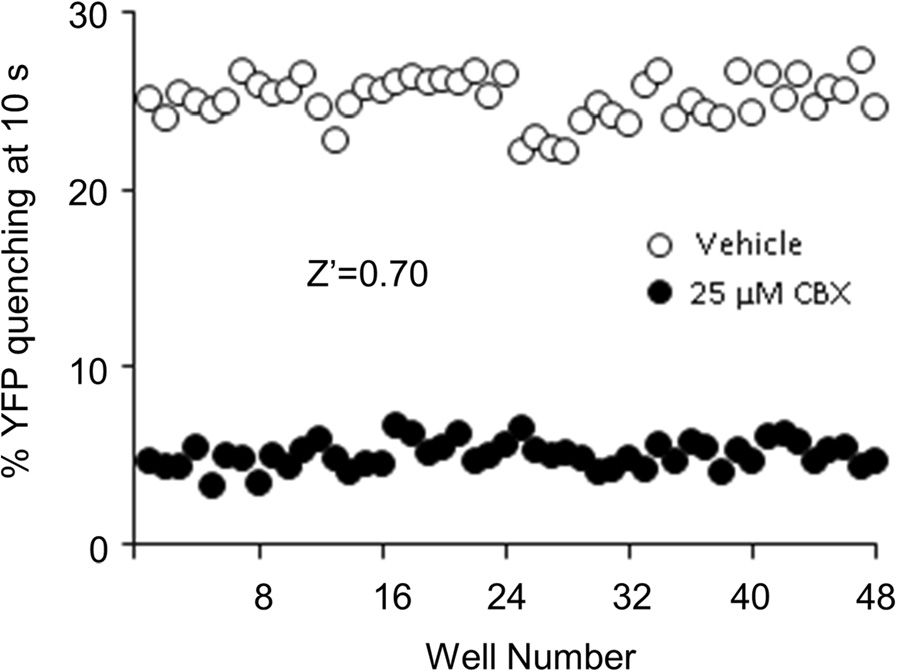
Application of this assay system to HOS cells. HOS-I^−^ and HOS-YFP^QL^ cells were mixed at a 2:1 ratio and plated on a 96-well plate. After 24 h of incubation, the medium was changed to C-solution containing vehicle or 25 μM CBX, followed by further incubation for 10 min before the 10-s I^−^-YFP assay. The percent YFP quenching at 10 s was plotted against for each well. Z’ factor was calculated as described in the Methods section and shown in the graph

To determine whether this GJIC assay was applicable to other cell types, HOS human osteosarcoma cells were chosen due to their expression of connexin43 (Additional file 1). As in the LN215-I^−^/LN215-YFP^QL^ system, a 2:1 mixture of HOS-I^−^ and HOS-YFP^QL^ cells was plated 24 h before treatment with vehicle or 25 μM CBX. Each group contained 48 wells treated for 10 min. The I^−^-YFP^QL^ assay was performed well by well. The percent YFP quenching at 10 s is shown in Fig. 5. The Z’ factor was 0.70.

## Discussion

In 2003, Aventis published an HTS system for a GJ modulator [18] using a high content screening system. They co-plated donor cells preloaded with calcein-AM and receptor cells on multi-well plates and chased the fluorescence image of each well. Calcein diffusion from donor cells to neighboring receptor cells was quantified by specialized image analysis software. Although calcein-AM used in GJIC assay becomes cell-impermeable after its acetomethoxy group is removed by intracellular esterases, calcein can be actively pumped out by multidrug resistance protein 1 [19]. Thus, donor cells should be preloaded with calcein-AM, and donor and acceptor cells should be detached, mixed and plated, treated with drug, and incubated to allow for dye diffusion through the GJs with time-lapse imaging sequentially without pause. This long, laborious method is difficult to schedule and is error-prone. Cells that need a long time to attach to the culture surface and form GJs are not appropriate for this assay system. Recently another HTS system for GJ modulators was developed by GlaxoSmithKline R&D [20]. They measured the propagation of cellular calcium signal using a Ca^2+^-sensitive, bioluminescent protein, aequorin. This assay system also used donor and acceptor cells. Donor cells were engineered to express the adrenergic α_1_ receptor or TrpV1 ion channel to generate a calcium signal. This system quantifies light emitted from acceptor cells, which express codon-optimized aequorin. The aequorin prosthetic group, coelenterazine, must be added to culture. Assaying the propagation of a calcium signal, an important physiological event mediated by GJ, is a strong benefit of this system. However, it can also be a drawback because any modulator of calcium signalling can be detected, most of which may be false positives. The adrenergic α_1_ receptor or TrpV1 channel can also cause false positives.

We developed an improved HTS for GJ modulators. An iodide transporter, SLC26A4, and a highly sensitive iodide sensor, YFP^QL^, were expressed by lentiviral vectors in donor and acceptor cells, respectively. Iodide transport from donor to acceptor cells via GJs was measured by YFP^QL^ quenching. While coelenterazine, a prosthetic group of aequorin is necessary for the aequorin assay, no additional reagent is needed for the YFP^QL^ fluorometry, which lowers the cost of this assay. Another merit of I^−^-YFP^QL^ assay is the short assay time. When we used LN215 or HOS cells, 10 s was enough to achieve excellent assay performance. Furthermore if the Z’ factor of the I^−^-YFP^QL^ GJIC assay is low in another cell type, extending the assay time can improve the performance, as shown in Fig. 4b. To our knowledge, iodide is not an important intracellular signalling molecule like Ca^2+^. Although using iodide might be a drawback of this assay, it actually reduces the chance of false-positives. Signal generators in the donor cells, adrenergic α_1_ receptor or TrpV1, of the Ca^2+^-aequorin assay and SLC26A4 in I^−^-YFP^QL^ assay can be affected by other chemicals, which creates the potential for false-positives. Cellular calcium signals have numerous targets and modulators, but, to date, no SLC26A4 modulators have been reported. Aequorin and YFP^QL^ sensors in acceptor cells are also pseudo-sources of false-positive results. The Ca^2+^-aequorin and I^−^-YFP^QL^ GJIC assays are compared in Table 1.

**Table 1:** Comparison of the Ca^2+^-aequorin assay and I^−^-YFP^QL^ assay

	Ca^2+^-aequorin assay [18]	I^−^-YFP^QL^ assay (this report)
Signal	Luminescence	Fluorescence
Reagent needed for assay	Coelenterazine	None
Assay time (sec per well)	40	10 Longer assay time increases Z’ factor.
Measuring physiologic event?	Yes (Ca^2+^)	No (I^−^)
Sources of false positives	● Adrenergic α_1_ receptor or TrpV1 channel in donor cells	● SLC26A4
	● Any target affecting cellular calcium	● Any target increasing cellular permeability to I^−^
	● Aequorin	● YFP^QL^

The I^−^-YFP^QL^ assay has the same limitations as other GJIC assays. False positives are possible, as are chemicals that do not affect GJIC activity but do change the YFP quenching rate. All GJ-inhibiting hits obtained from this assay must be confirmed to not inhibit SLC26A4. Cells expressing both SLC26A4 and YFP^QL^ (i.e., LN215-I^−^-YFP^QL^) can be used for this determination. Chemicals that weaken the YFP quenching by iodide in the cells act as false positives. All detected GJ activators must be confirmed to not enhance YFP^QL^ quenching by iodide when acceptor cells are plated alone. The Ca^2+^-aequorin assay can also be used as a second screening method to confirm positive results.

In theory, the I^−^-YFP^QL^ assay can measure the activity of any intercellular channels that allow the diffusion of iodide, despite their composition. Although pannexins have sequence homologies with innexins, the invertebrate GJ proteins, they have not been confirmed to form GJs [21]. A recent report by Sahu et al. showed that the formation of GJs by pannexins depends on cell type [22]. Undocked pannexin hemichannels were blocked by treatment with 30 μM CBX, but pannexin GJs were not. Thus, even if pannexin GJs exist in LN215 cells, the GJ inhibition by CBX observed in the present report was not due to the inhibition of pannexins.

We used lentiviral vectors to produce donor and acceptor cells in this study. An I^−^-YFP^QL^ GJIC assay system can be set up with other types of cells with functional GJs, by transducing the cells with the two viral vectors followed by antibiotic selection to create donor and acceptor cells. Because lentivirus integrates its genome into the host cell chromosomes with its integrase, most transduced cells express the transgenes stably. Thus, this system is easy to apply to other cell types. Cells that are impermeable to iodide are ideal for use in the proposed GJIC assay. If a cell line shows sufficiently greater YFP^QL^ quenching by iodide when donor and acceptor cells are mixed and plated than when only acceptor cells are plated, it can also be regarded as suitable for this GJIC assay.

## Conclusions

We developed a new GJIC assay for HTS, which detects iodide diffusion through GJs. The only materials needed for this assay are donor and acceptor cells, a multi-plate reader capable of fluorometry and automated injection, C-solution, and I-solution. No dyes or reagents to read luminescence or to generate a calcium signal are needed. Each assay can be performed in 10 s. When this system was used in LN215 human glioma cells and HOS human osteosarcoma cells, the Z’ factors were over 0.7. This assay is predicted to have a lower false-positive rate than that of the Ca^2+^-aequorin assay. Thus, this assay is appropriate for HTS to develop small molecule drugs targeting GJs.

## Methods

### Cell culture

LN215 human glioma cells (a kind gift from Dr. Erwin G. Van Meir), HOS human osteosarcoma cells (ATCC), and HEK293T cells (ATCC) were cultured in Dulbecco’s modified Eagle Medium (DMEM) containing 100 IU/ml penicillin, 100 μg/ml streptomycin, and 10 % fetal bovine serum. All cultures were maintained in humidified 5 % CO_2_/95 % air at 37 °C. Ethical approval or consent to the use of the cells used in this study is not required.

### Plasmid construction

The lentiviral plasmid expressing YFP^QL^, pLVX-EIP-YFP^QL^, was constructed by inserting a DNA fragment encoding YFP^QL^ into pLVX-EF1α-IRES-Puro purchased from Clontech. The YFP^QL^ fragment was prepared by a NheI/XbaI digestion of pcDNA3.1/Hygro(+)-YFP-H148Q/I152L (a kind gift from Wan Namkung) and inserted in the SpeI site of the backbone plasmid.

To construct the lentiviral plasmid expressing SLC26A4, an empty lentiviral vector, pLenti6P, was generated, and SLC26A4 was inserted. The construction procedures are described below. A blunted woodchuck posttranscriptional regulatory element (WPRE) was prepared by ClaI digestion of pSUB/AM-CBA-EGFP-WPRE-bGH [23] and subsequent blunting with Klenow fragment. The blunted WPRE was inserted at the blunted NsiI site of pLKO.1puro (Sigma). The resulting plasmid, pLKO-W, had puromycin acetyltransferase (PAC) linked to WPRE. A lentiviral plasmid from Life Technologies, pLenti6/V5-GW/LacZ, was modified by EcoRV digestion followed by self-ligation, and the product was named pLenti6. A PAC-WPRE fragment with 5’-blunt and 3’-KpnI was prepared from pLKO-W by sequential BamHI digestion, blunting, and KpnI digestion. This PAC-WPRE fragment was inserted at a SmaI/KpnI site of pLenti6, leading to an empty vector, pLenti6P, that expressed PAC as a selection marker and contained WPRE to enhance transgene expression. SLC26A4 was removed from pcDNA3.1(+)-hSLC26A4 (a kind gift from Min Goo Lee) by NheI/XbaI digestion and inserted at the XbaI site of pLenti6P.

### Lentivirus production

HEK293T cells were plated on six-well plates at a density of 4 * 10^5^ cells/well and incubated for 24 h. Lentiviral plasmid, psPAX2, and pMD2.G were mixed at a ratio of 4:3:1, and 3 μg of the mixture was transfected into HEK293T cells with Lipofectamine2000 (Invitrogen) for 15 h. Cultures were refreshed with 2 mL of growth media and cultivated for another 36 h. Media containing lentivirus was harvested and cleared by centrifugation at 3000 rpm for 3 min before storage at −80 °C.

### Lentiviral transduction and selection

LN215 and HOS cells were plated on a 24-well plate at 30 % confluence. After 24 h, the cells were transduced with 400 μL of a 1:1 mixture of lentivirus and fresh growth medium supplemented with 4 μg/mL polybrene (Sigma) for 15 h before media change. Cells were further incubated for 72 h and detached to be re-plated on six-well plates with 2 μg/mL puromycin. After a week of puromycin selection, cells were used for the GJIC assay.

### I^−^-YFP^QL^ GJIC assay

Donor and receptor cells were detached with trypsin, counted, mixed, and plated on 96-well plates. After 24 h of incubation, culture media were changed to 100 μL of C-solution (10 mM HEPES, pH 7.4, 140 mM NaCl, 10 mM glucose, 5 mM KCl, 1 mM MgCl_2_, 1 mM CaCl_2_). Vehicle or 25 μM CBX was used to treat cultures for 10 min when drug treatment was indicated. The GJIC assay was performed well by well. The fluorescence of each well was measured with a POLARstar microplate reader (BMG Labtech, Germany) every 0.4 s for the indicated time. One second after the first measurement, 100 μL of I-solution (10 mM HEPES, pH 7.4, 140 mM NaI, 10 mM glucose, 5 mM KCl, 1 mM MgCl_2_, 1 mM CaCl_2_) was added via the injector in the plate reader. The percent YFP^QL^ fluorescence to time zero was calculated. The YFP^QL^ quenching rate at a selected time point reflected GJ activity.

### Calculation of Z’ factor

Z’ factor has been used as a quality indicator of HTS systems [17]. Z’ factor was calculated as follows:

$$ \mathrm{Z}\mathit{\hbox{'}}=1-\frac{3{\sigma}_{c+}+3{\sigma}_{c-}}{\left|{\mu}_{c+}+{\mu}_{c-}\right|}, $$

where σ and μ represent standard deviation and mean, respectively, and c + and c- are the positive and negative controls.

## Additional files

Additional file 1:
**Expression of connexin43 in LN215 and HOS cells.** To determine whether LN215 and HOS cells express a major connexin, connexin43, we performed immunoblot analysis with anti-connexin43 antibody (C13720, Transduction Laboratories). HeLa, LN215 and HOS cells were lysed with PBS containing 1 % Triton X-100 and cOmplete™ protease inhibitor cocktail (Roche) before centrifugation at 15,000 × g for 10 min at 4 °C. After the BCA protein assay, 20 μg of protein was used for immunoblotting. Since HeLa cells do not express connexin43 [24], they were used as a negative control. Connexin43 (arrow head) was detected in LN215 and HOS lysates. (TIFF 271 kb) Additional file 2:
**Determination of the optimal ratio of donor and acceptor cells.** This is the raw data (fluorescence intensity reads) in Fig. 3a (Microsoft Excel format). (XLS 51 kb) Additional file 3:
**Determination of the optimal ratio of donor and acceptor cells.** This is another presentation of the data in Fig. 3a. The fluorescence intensity reads from the seven groups were presented in individual graphs without normalization or background subtraction. Final quenching rates were also shown. Rapid fluorescence reduction before 2 s was due to media dilution by added I-solution. (TIFF 1223 kb) Additional file 4:
**Effect of CBX on YFP quenching when only LN215-YFP** ^**QL**^ **cells were plated.** Only LN215-YFP^QL^ cells or a 2:1 mixture of LN215-I^−^ and LN215-YFP^QL^ cells were plated on 96-well plate 24 h before treatment with vehicle or 25 μM CBX for 10 min. Each treatment was added to three wells. The I^−^-YFP^QL^ assay was performed for 10 s. The mean % YFP fluorescence ± standard deviation was plotted against time. (TIFF 289 kb) Additional file 5:
**List of chemicals tested in this study.** (TIFF 397 kb)

## Abbreviations

2-APB: 2-aminoethoxydiphenyl borate
AM: Acetoxymethyl ester
CBX: Carbenoxolone
GJ: Gap junction
GJIC: Gap junctional intercellular communication
HTS: High-throughput screening
PAC: Puromycin acetyltransferase
WPRE: Woodchuck posttranscriptional regulatory element
YFP^QL^: Yellow fluorescent protein H148Q/I152L
