# Supplementary figures and images for: A new high-throughput screening-compatible gap junctional intercellular communication assay
Source: BMC Biotechnol. 2015 Oct 6;15:90. doi: 10.1186/s12896-015-0211-3 (PMC4596302; doi:10.1186/s12896-015-0211-3)

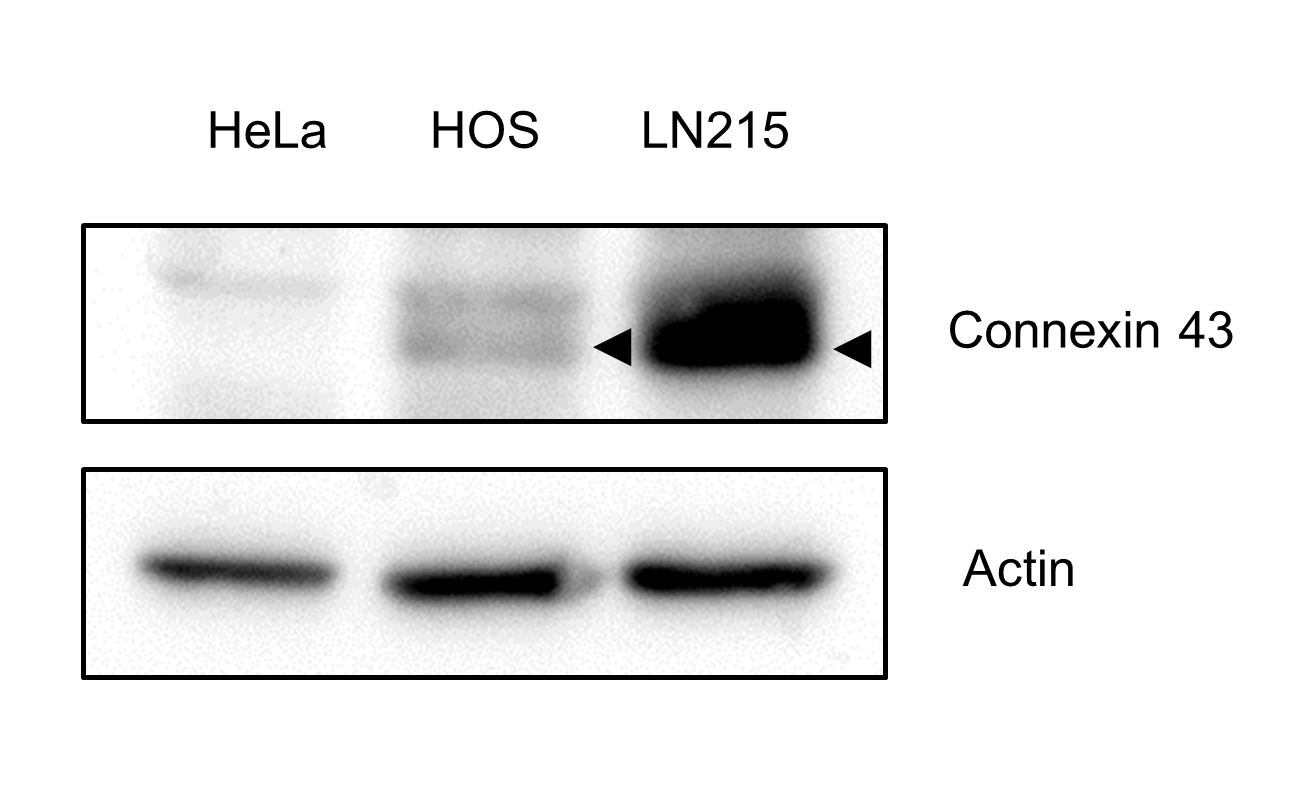

Supplement: Additional file 1: — Expression of connexin43 in LN215 and HOS cells. To determine whether LN215 and HOS cells express a major connexin, connexin43, we performed immunoblot analysis with anti-connexin43 antibody (C13720, Transduction Laboratories). HeLa, LN215 and HOS cells were lysed with PBS containing 1 % Triton X-100 and cOmplete™ protease inhibitor cocktail (Roche) before centrifugation at 15,000 × g for 10 min at 4 °C. After the BCA protein assay, 20 μg of protein was used for immunoblotting. Since HeLa cells do not express connexin43 [24], they were used as a negative control. Connexin43 (arrow head) was detected in LN215 and HOS lysates. (TIFF 271 kb) [file 12896_2015_211_MOESM1_ESM.tiff]

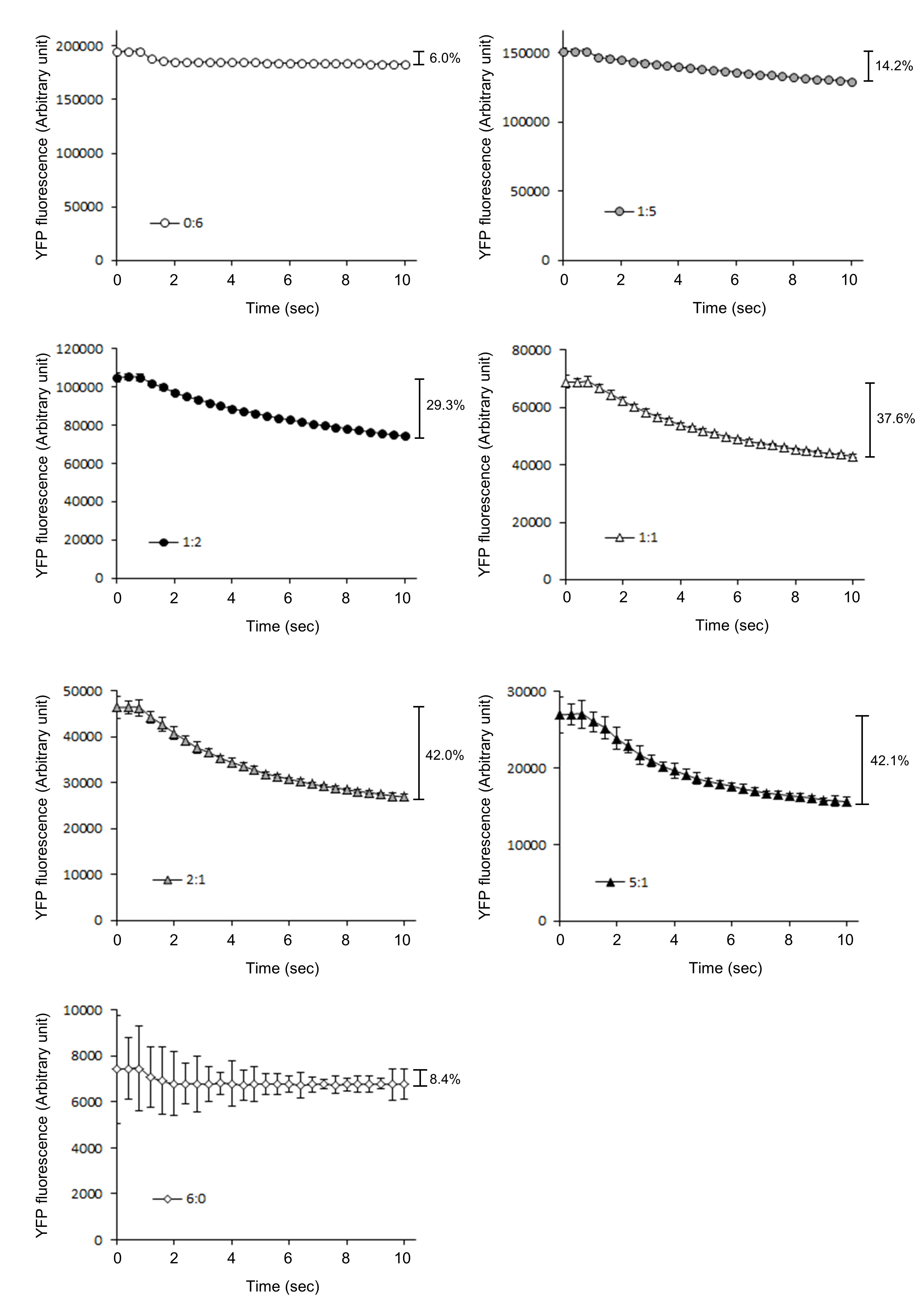

Supplement: Additional file 3: — Determination of the optimal ratio of donor and acceptor cells. This is another presentation of the data in Fig. 3a. The fluorescence intensity reads from the seven groups were presented in individual graphs without normalization or background subtraction. Final quenching rates were also shown. Rapid fluorescence reduction before 2 s was due to media dilution by added I-solution. (TIFF 1223 kb) [file 12896_2015_211_MOESM3_ESM.tiff]

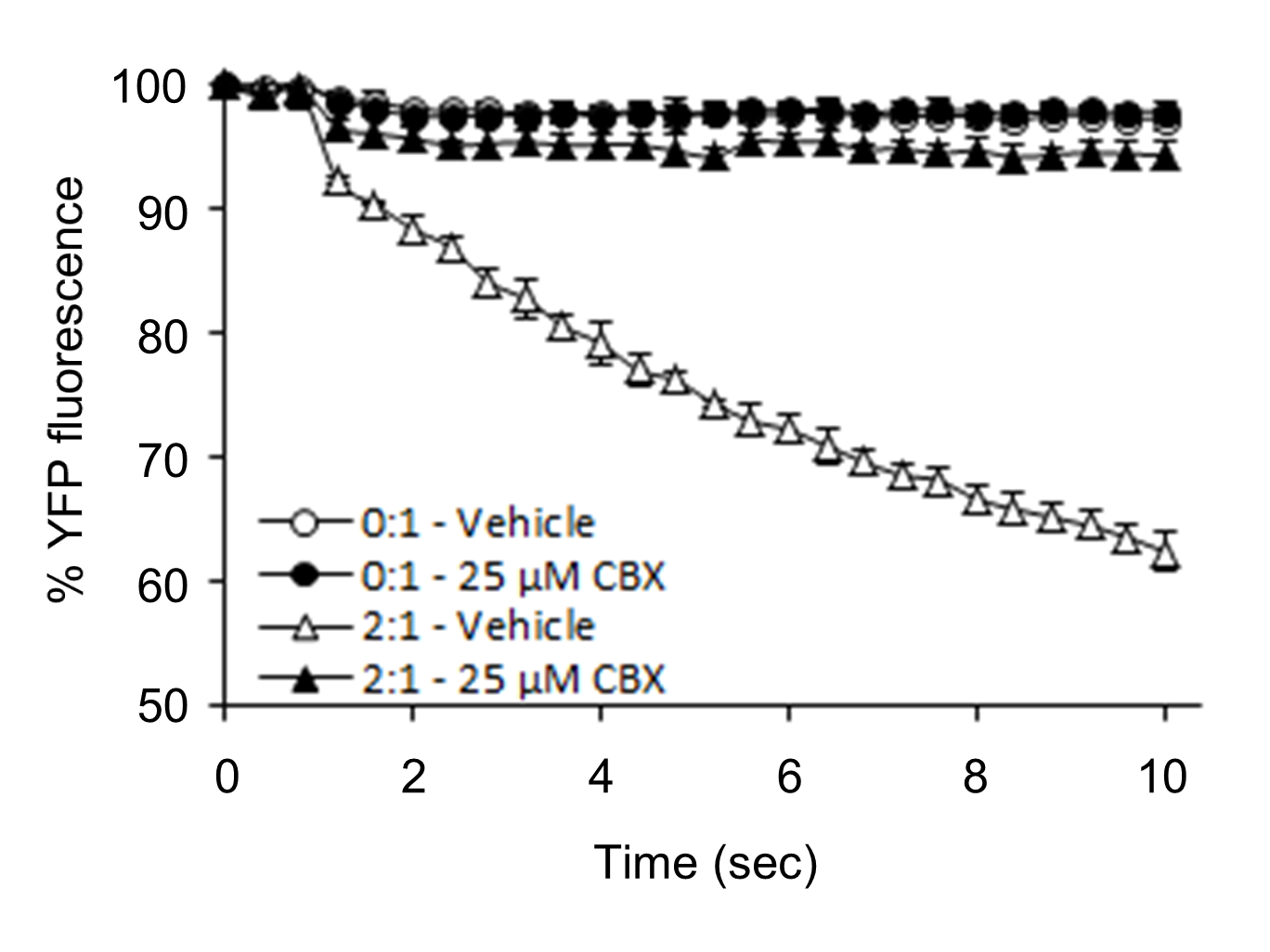

Supplement: Additional file 4: — Effect of CBX on YFP quenching when only LN215-YFP QL cells were plated. Only LN215-YFPQL cells or a 2:1 mixture of LN215-I− and LN215-YFPQL cells were plated on 96-well plate 24 h before treatment with vehicle or 25 μM CBX for 10 min. Each treatment was added to three wells. The I−-YFPQL assay was performed for 10 s. The mean % YFP fluorescence ± standard deviation was plotted against time. (TIFF 289 kb) [file 12896_2015_211_MOESM4_ESM.tiff]

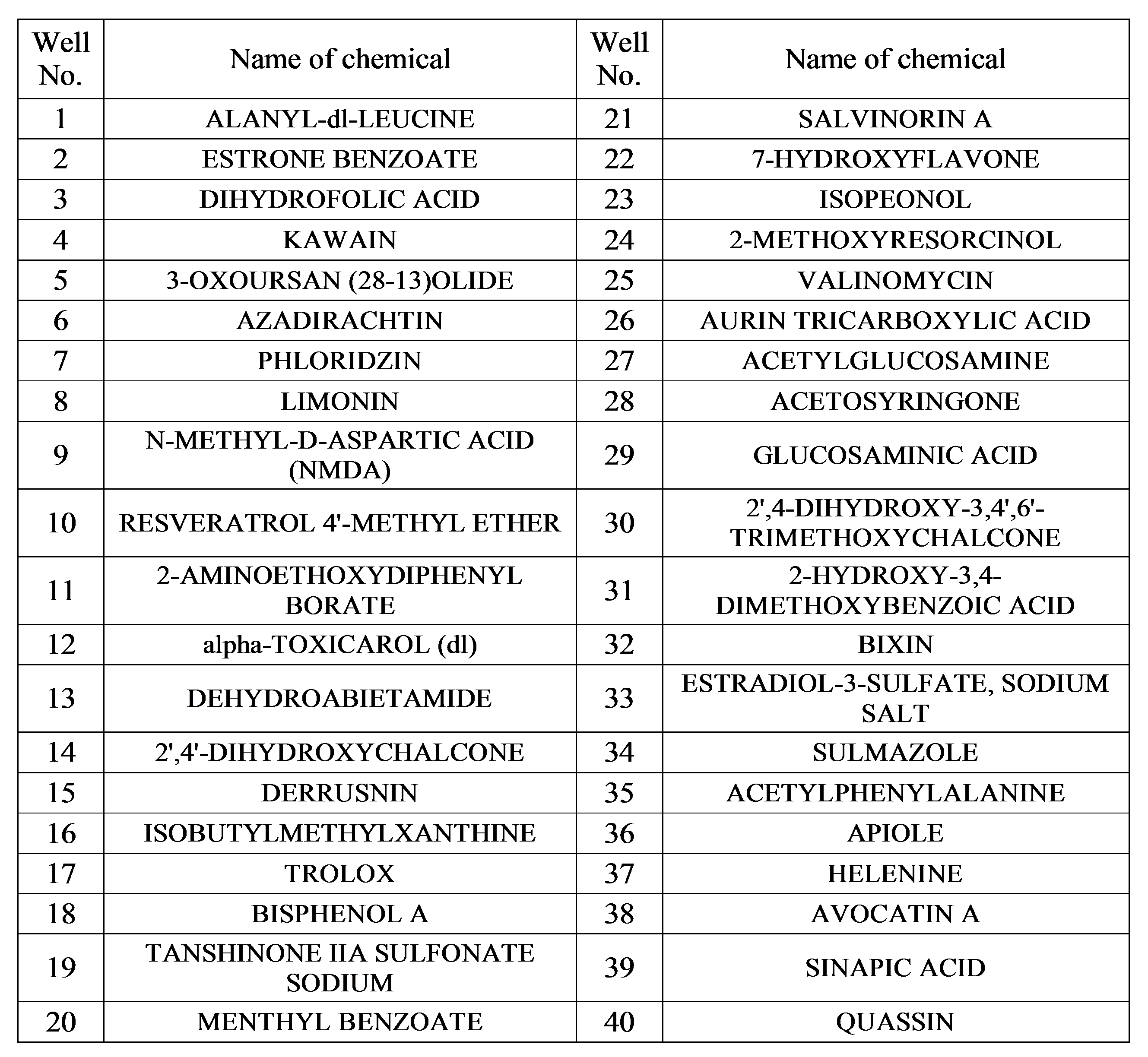

Supplement: Additional file 5: — List of chemicals tested in this study. (TIFF 397 kb) [file 12896_2015_211_MOESM5_ESM.tiff]
